# Can Bone Morphogenetic Protein 1 (BMP1) Be a Potential Biomarker of Obesity?

**DOI:** 10.7759/cureus.67025

**Published:** 2024-08-16

**Authors:** Emel Saglam, Hande Karagedik, Mustafa Dinc, Deniz Oke, Palmet Gun Atak, Burcak Karadeniz, Gokhan Burul, Uzay Gormus Degrigo

**Affiliations:** 1 Internal Medicine, Bagcilar Training and Research Hospital, Istanbul, TUR; 2 Molecular Medicine, Aziz Sancar Institute for Experimental Medicine, Istanbul, TUR; 3 Endocrinology and Metabolism, Kirklareli Training and Research Hospital, Kirklareli, TUR; 4 Physical Medicine and Rehabilitation, Gaziosmanpasa Training and Research Hospital, Istanbul, TUR; 5 Biochemistry, Vadi Istanbul Liv Hospital, Istanbul, TUR; 6 Rheumatology, Bagcilar Training and Research Hospital, Istanbul, TUR; 7 Biochemistry and Biophysics, Karolinska Institute, Stockholm, SWE

**Keywords:** bone morphogenetic protein 1, neuregulin 4, quicki, obesity, apolipoprotein a5

## Abstract

Background

Obesity has long been a severe threat to public health as an epidemic, and studies on its pathogenesis and treatment have been ongoing. Our study aims to compare the serum levels of bone morphogenetic protein 1 (BMP1), neuregulin 4 (NRG4), and apolipoprotein A5 (ApoA5) in obese and non-obese individuals and investigate their association with obesity.

Methodology

Our study included a total of 111 participants, of whom 46 were obese (body mass index (BMI) ≥30 kg/m^2^), aged 18-65 years, and had no comorbidities, and 65 were non-obese (BMI = 18.5-29.9 kg/m^2^) without any additional disease. For all participants, BMP1, NRG4, and ApoA5 levels were determined and compared with clinical and biochemical parameters.

Results

Overall, 60.4% (n = 67) of the participants were female and 39.6% (n = 44) were male. In terms of the BMI scores, 58.6% (n = 65) had a BMI <30 kg/m^2^ and 41.4% (n = 46) had a BMI ≥30 kg/m^2^. Both, the BMI and the gender groups did not differ significantly in terms of age (p = 0.093 and p = 0.795, respectively). The weight, fat-free mass, mineral quantity, protein quantity, fluid weight, and fluid ratio values of the male participants were significantly higher than females (p = 0.011, p = 0.001, p = 0.001, p = 0.001, p = 0.001, and p = 0.001, respectively). The aspartate aminotransferase (AST)/alanine aminotransferase (ALT) ratios and the triglyceride/glucose (TG/Glu) ratios were found to be significantly higher in males than in females (p = 0.001 and p = 0.001, respectively). The respective BMP1 (15.88 vs. 13.35), AST/ALT (1.36 vs. 1.04) and TG/Glu ratios (1.47 vs. 1.29) were significantly higher, while the quantitative insulin sensitivity check index (QUICKI) was lower in obese individuals than in non-obese individuals (0.32 vs. 0.34). NRG4 and ApoA5 values were similar between the two groups. BMP1, QUICKI values, and AST/ALT ratios proved to be statistically significant in obesity through the univariable logistic regression analysis (β = 1.066, p = 0.048; β = 0.0001, p = 0.001, and β = 3.707, p = 0.003, respectively). On multiple logistic regression analysis, QUICKI values (β = 0.001, p = 0.001) had a negative and significant effect on obesity, and the AST/ALT ratios (β = 2.803, p = 0.033) had a positive and significant effect on obesity.

Conclusions

Our study indicates that detecting an important link between BMP1 in obese patients will help elucidate the pathogenesis of obesity and come up with a potential therapeutic candidate. BMP1 levels, along with AST/ALT and TG/Glu ratios, were significantly higher in obese patients. BMP1 levels were also an independent significant predictor of obesity together with AST/ALT ratio and QUICKI in this study, suggesting that it may exhibit a metabolic deterioration in obese individuals. However, the results cannot absolutely tell whether it supported deterioration or was a component of the repair mechanism. Althoughit is generally known from recent studies that BMP1 plays a role in osteogenesis, some encouraging results were obtained in our study indicating that BMP1 may play a role in the pathogenesis of obesity. It is expected that our results will not only promote the elucidation of the pathogenesis of obesity, but also provide a therapeutic agent.

## Introduction

Although the body mass index (BMI) cut-off value varies depending on ethnicity, obesity, which is technically accepted as a BMI over 30 kg/m^2^, is an important public health problem that is rapidly increasing in Turkey and globally. In a nationwide study conducted in Turkey, the prevalence of obesity was found to be 36%, indicating a 40% increase in obesity and 35% increase in central obesity compared to a study performed 12 years earlier [1].

Adipocytes are the predominant cell types of adipose tissue in the body, and a large store of excess energy as fat [2]. Under physiological and pathological conditions, they are a crucial resource for many metabolites, cytokines, and adipokine hormones [3]. Obesity affects the regulation of lipid metabolism by causing hypertrophy, hyperplasia, and inflammation in adipocytes, along with various changes in the secretion of adipokines [4]. In previous studies, the volume of adipocytes isolated from obese mice with various metabolic disorders had significantly increased [5,6]. In humans, adipocyte dysfunction is predicted to have a significant pathophysiological role in obesity, as well as associated chronic metabolic conditions [7].

Bone morphogenetic proteins (BMPs) belong to a similar superfamily that also includes transforming growth factor-β (TGF-β), growth and differentiation factors, and activins [8]. Although initially realized for their ability to start bone formation, BMPs are now known to have an impact on all organ systems, such as the development and maintenance of adult tissue homeostasis [9]. Mesenchymal stem cells can differentiate through various lineages. The certain lineage is regulated partially by BMPs. Obesity is identified by the deposition of white adipose tissue as a result of an increase in adipocyte size and/or adipocyte count [9]. Adipose tissue mesenchymal stem cells are a kind of reservoir and can turn into adipocytes under suitable conditions. Some members of BMPs have been shown to promote the formation of adipocytes from adipose precursor cells. Although BMP9 is an osteogenic BMP that is a potent inducer of osteocyte differentiation, BMP2 and BMP4 can induce pluripotent stem cells in adipocytes [10]. BMP4 and BMP7 are the most widely studied BMPs that have been proven to have an impact on white and brown adipogenesis, respectively, and BMP2, BMP6, and BMP8b also have activity in adipogenesis [8]. Resistance to BMP4 by Gremlin 1, which is an inhibitor of both BMP4 and BMP7, has been reported to result in the characterization of hypertrophic obesity [11]. BMP1 is a metalloprotease that can induce bone and cartilage development, and does not belong to the TGF-β superfamily [12]. To date, studies on BMP1 have focused mainly on osteogenesis, with its role in bone formation and organogenesis still unclear [13], and its role in adipogenesis is yet to be sufficiently examined.

Neuregulin 4 (NRG4) has just recently been accepted as a member of the adipokine family. NRG4 is released from brown adipose tissue secretomes during brown adipose tissue differentiation in mice [14]. NRG4 belongs to the epidermal growth factor (EGF) family, with its extracellular ligands being involved in activating ErbB3 and ErbB4 tyrosine kinase receptors [15]. In a study, low serum NRG4 concentrations were found to be related to insulin resistance (IR), fatty liver disease, as well as obesity in obese children [16]. It was shown earlier that adipose tissue NRG4 expression decreased in both obese animal models and obese human adults [15]. On the other hand, in recent years, apolipoprotein A5 (ApoA5), a newly discovered member of the apolipoprotein superfamily, has been closely associated with obesity and the development of metabolic syndrome and has a significant impact on regulating triglyceride metabolism [17]. The fact that the plasma level of ApoA5 is consistently low in obese individuals and that it is inversely correlated with BMI supports the relationship between ApoA5 levels and the pathophysiology of obesity [18,19]. In particular, ApoA5 modulates the excessive accumulation of triglycerides in adipose tissue, which is the hallmark of obesity [20].

Our study aims to compare the relationship between the demographic characteristics and the biochemical parameters of the participants and biomarkers (BMP1, NRG4 and ApoA5) thought to be related to obesity. In this study, we investigated the roles of the parameters that we believe play a role in the disease mechanism utilizing the results determined in obese patients and the possibility of the relationship between them as a biomarker.

## Materials and methods

Study design and participants

The study included a total of 111 participants between the ages of 18 and 65 years who presented to our clinic with obesity. The age and gender of the participants were recorded. The obesity status was determined according to the following formula: BMI = weight (kg)/height (m)^2^. The Tanita body composition analyzer (Tanita Corporation of America, Illinois, USA) was used to measure the height and weight of the patients. The degree of obesity (the distance to the most optimal calculated weight as %), fat mass, mineral quantity, protein quantity, fluid weight, fluid ratio, fat weight, and fat ratio parameters were recorded.

Patients with one or more of the following conditions that may affect metabolic parameters were excluded from the study: hyper/hypothyroidism, renal failure, liver diseases, heart failure, alcoholism, malignancy, pregnancy, pancreatic diseases, diabetes mellitus (DM), and other chronic diseases.

Clinical classification

Obsesity is defined as an excessive accumulation or abnormal distribution of body fat. The National Institute of Health (NIH) classifies BMI as normal weight (BMI = 18.5-24.9 kg/m^2^), overweight (BMI = 25-29.9 kg/m^2^), or obese (BMI ≥30 kg/m^2^). Accordingly, a total of 46 patients with obesity (BMI ≥30.0 kg/m^2^) who did not have any chronic disease and 65 non-obese patients (BMI = 18.5-29.9 kg/m^2^) with no additional disease were included.

Biochemical analysis

All biochemical tests were conducted in the morning following an 8 to 10-hour fasting. Routine biochemical analyses of the participants were studied in the Biochemistry Laboratory of the Sisli Florence Nightingale Hospital of Istanbul, Bilim University. Glucose, total cholesterol, triglyceride (TG), high-density lipoprotein cholesterol (HDL-C), low-density lipoprotein cholesterol (LDL-C), aspartate aminotransferase (AST), alanine aminotransferase (ALT), uric acid, and creatinine analyses were performed using the Roche modular system Cobas E 501 device by spectrophotometric method. Insulin analyses were performed using the Roche modular system Cobas E 601 device by the electrochemiluminescence immunoassay method. The previously separated serum samples were centrifuged at a maximum speed of 2,500× for 10 minutes and stored at -80°C for BMP1, NRG4, and ApoA5 serum determination. BMP1, NRG4, and ApoA5 were determined using the BT Labs enzyme-linked immunosorbent assay (ELISA) kit (catalog number: E2240Hu BMP1, standard curve range: 5-1,000 ng/L, sensitivity: 2.67 ng/L; catalog number: E2292Hu NRG4, standard curve range: 1-400 ng/L, sensitivity: 0.54 ng/L; catalog number: E1968Hu ApoA5, standard curve range: 7-1,500 ng/L, sensitivity: 3.13 ng/L; precision: CV (%) = SD/mean × 100, precision: intra-assay: CV <8%, inter-assay: CV <10%) in Multiscan Spectrum (Thermo Electron Corporation, USA) with three samples taken from each patient. The serum samples were investigated using the ELISA method in the Istanbul Bilim University Clinical Research laboratory.

To determine the degrees of IR and insulin sensitivity (IS), the homeostasis model assessment (HOMA) and the quantitative insulin sensitivity check index (QUICKI) were used, respectively. HOMA-IR was calculated using the following formula: fasting insulin (μU/mL) × fasting glucose (mg/dL)/405] [21]. QUICKI was calculated using the following formula: [1/(log (fasting insulin (μU/mL)) + log (fasting glucose (mg/dL)] [22].

Statistical analysis

The Number Cruncher Statistical System (NCSS) 2007 (Kaysville, Utah, USA) was used for statistical analysis. The Shapiro-Wilk test was used to evaluate descriptive statistical methods (mean, standard deviation, median, frequency, ratio, minimum, maximum) and the data distribution. The Mann-Whitney test was used to compare the quantitative data between two groups not normally distributed. Student’s t-test was utilized to compare the quantitative data between two normally distributed groups. The receiver operating characteristic (ROC) analysis was performed to determine the specificity and sensitivity of BMP1 (p < 0.05). Univariable and multivariable logistic regression analysis was performed to determine the factors affecting the dependent variable.

Ethical considerations

Before conducting the study, ethical clearance was obtained from the Clinical Research Ethics Committee of Istanbul Bilim University (protocol number: 12.04.2016/48-01). The study was designed as a cross-sectional study. Written informed consent was obtained from all participants.

## Results

In this study, 60.4% (n = 67) of the participants were female and 39.6% (n = 44) were male. Regarding BMI, 58.6% (n = 65) of the participants had a BMI <30 kg/m^2^ and 41.4% (n=46) had a BMI ≥30 kg/m^2^. The BMI and gender groups did not differ significantly with age (p = 0.093 and p = 0.795, respectively). On the other hand, the groups were significantly different concerning anthropometric measurements (Table 1).

**Table 1:** Comparison of the anthropometric measurements by gender. ^a^: Student’s t-test (mean ± SD), ^b^: Mann-Whitney test (minimum-maximum/median). P-values <0.05 were considered statistically significant.

	Gender	n	Mean ± SD	Minimum–Maximum (median)	P-value
Height (cm)	Male	44	175.59 ± 6.33	164–200 (176)	0.209^a^
	Female	67	162.93 ± 6.12	147–178 (164)	
Weight (kg)	Male	44	90.47 ± 24.58	55–170 (84.15)	0.011^b^
	Female	67	78.3 ± 17.68	46.8–120 (76.4)	
Obesity degree (%)	Male	40	31.37 ± 29.3	-10.2–131.58 (23.54)	0.670^b^
	Female	60	32.89 ± 28.41	-9.2–109.71 (28.85)	
Fat-free mass (%)	Male	44	75.46 ± 9.18	50–93.4 (76.45)	0.001^b^
	Female	67	63.64 ± 8.37	45.2–86 (62)	
Mineral quantity (%)	Male	44	5.27 ± 0.8	3.27–6.7 (5.46)	0.001^a^
	Female	67	4.45 ± 0.7	3–6.3 (4.44)	
Protein quantity (%)	Male	44	14.96 ± 1.93	9.8–20.2 (15)	0.001^b^
	Female	67	12.54 ± 1.83	8.35–18 (12)	
Fluid weight (kg)	Male	44	48.63 ± 7.49	37.6–72.5 (47.5)	0.001^b^
	Female	67	35.75 ± 4.33	27–47 (35)	
Fluid ratio (%)	Male	44	55.26 ± 6.69	36.85–68.3 (55.96)	0.001^b^
	Female	67	46.8 ± 5.93	36.01–63.14 (45,32)	
Fat weight (kg)	Male	44	24.23 ± 16.01	3.6–84.8 (20.4)	0.007^b^
	Female	67	29.49 ± 12.39	6.7–57 (28.7)	
Fat ratio (%)	Male	44	24.49 ± 9.16	6.6–49.7 (23.55)	0.001^b^
	Female	67	36.07 ± 8.1	13.6–50.8 (38)	

The weight, fat-free mass, mineral quantity, protein quantity, fluid weight, and fluid ratio values of the male participants were significantly higher than the female participants (p = 0.011, p = 0.001, p = 0.001, p = 0.001, p = 0.001, and p = 0.001, respectively). However, the fat weight and fat ratio values of the males were lower than those of females (p = 0.007 and p = 0.001, respectively). Height and obesity values did not differ statistically according to gender (p = 0.209 and p = 0.670, respectively) (Table 1).

The BMP1, NRG4, ApoA5, and QUICKI values and MPV/PLT ratio were similar between males and females (p = 0.065, p = 0.906, p = 0.526, p = 0.501, and p = 0.291, respectively). The AST/ ALT ratios as well as the triglyceride/glucose indices (TG/Glu) were found to be significantly higher in males than in females (p = 0.001 and p = 0.001, respectively) (Table 2).

**Table 2:** Comparison of the laboratory parameters by gender. ^a^: Mann-Whitney test (minimum-maximum/median); ^b^: Student’s t-test (mean ± SD). P-values <0.05 were considered statistically significant. BMP1 = bone morphogenetic protein 1; NRG4 = neuregulin 4; ApoA5 = apolipoprotein A5; AST/ALT = aspartate aminotransferase/alanine aminotransferase ratio; TG/Glu = triglyceride/glucose index; QUICKI = quantitative insulin sensitivity check index

	Gender	n	Mean ± SD	Minimum-Maximum (median)	P-values
BMP1 (pg/mL)	Male	44	15.85 ± 6.12	7.80–25.60 (12.3)	0.065^a^
	Female	67	13.48 ± 5.69	6.80–26.30 (10.96)	
NRG4 (ng/mL)	Male	44	3.61 ± 4.32	1.03–26.90 (2.1)	0.906^a^
	Female	67	4.12 ± 4.95	1.01–23.60 (2)	
ApoA5 (pg/mL)	Male	44	118.10 ± 188.32	9.80–1,067.70 (65.05)	0.526^a^
	Female	67	134.48 ± 208.50	7.10–959.50 (55.60)	
AST/ALT ratio	Male	44	1.4 ± 0.61	0.5–3.16 (1.33)	0.001^a^
	Female	67	1.02 ± 0.38	0.24–2.48 (0.95)	
MPV/PLT ratio	Male	44	0.04 ± 0.01	0–0.08 (0.04)	0.291^a^
	Female	67	0.04 ± 0.01	0–0.08 (0.04)	
TG/Glu	Male	44	1.73 ± 1.21	0.52–7 (1.45)	0.001^a^
	Female	67	1.13 ± 0.57	0.46–2.91 (0.97)	
QUICKI	Male	44	0.34 ± 0.03	0.29–0.43 (0.33)	0.501^a^
	Female	67	0.33 ± 0.03	0.27–0.41 (0.33)	

The BMP1 levels and AST/ALT and TG/Glu ratios of obese subjects proved to be significantly higher than those of non-obese (p = 0.018, p = 0.001, and p = 0.023, respectively). The NRG4, ApoA5, and MPV/PLT values did not differ significantly between obese and non-obese subjects (p = 0.220, p = 0.999, and p = 0.726, respectively). Glucose, insulin, HOMA-IR, uric acid, creatinine, ALT, and triglyceride values of non-obese individuals proved to be lower than those of obese (p = 0.019, p = 0.001, p = 0.001, p = 0.001, p = 0.056, p = 0.001, and p = 0.012, respectively). While the QUICKI values (which reflect IS) of the obese were lower than those of the non-obese subjects (p = 0.001), the HDL values of non-obese subjects were significantly higher than those of obese subjects (p = 0.008). Age, total cholesterol, LDL-C, and AST values did not differ statistically according to the BMI (p = 0.093, p = 0.476, p = 0.611, and p = 0.142, respectively) (Tables 3, 4).

**Table 3:** Comparison of the laboratory parameters according to BMI. ^a^: Mann-Whitney test (minimum-maximum/median); ^b^: Student’s t-test (mean ± SD). P-values <0.05 were considered statistically significant. BMI = body mass index; BMP1 = bone morphogenetic protein 1; NRG4 = neuregulin 4; ApoA5 = apolipoprotein A5; UA = uric acid; AST = aspartate aminotransferase; ALT = alanine aminotransferase; MPV = mean platelet volume; PLT = platelet

	Obesity status	n	Mean ± SD	Minimum-Maximum (median)	P-value
BMP1 (pg/mL)	Non-obese	65	13.35 ± 5.68	6.80–26.30 (10.6)	0.018^a^
	Obese	46	15.88 ± 6.07	6.90–25.6 (12.3)	
NRG4 (ng/mL)	Non-obese	65	4.31 ± 5.34	1.02–26.90 (2.1)	0.220^a^
	Obese	46	3.33 ± 3.55	1.04–15.70 (1.9)	
ApoA5 (pg/mL)	Non-obese	65	107.82 ± 147.6	7.1–651.9 (64.80)	0.999^a^
	Obese	46	156.48 ± 255.7	8.3–1,067.7 (53.95)	
UA (mg/dL)	Non-obese	65	4.69 ± 1.31	2.4–9.1 (4.5)	0.001^a^
	Obese	46	5.55 ± 1.37	3.1–9.7 (5.65)	
Creatinine (mg/dL)	Non-obese	65	0.75 ± 0.21	0.5–1.8 (0.7)	0.056^a^
	Obese	46	0.8 ± 0.17	0.5–1.1 (0.8)	
AST (U/L)	Non-obese	65	20.08 ± 10.36	12–62 (17)	0.142^a^
	Obese	46	21.96 ± 9.87	11–57 (18)	
ALT (U/L)	Non-obese	65	23.58 ± 24.77	4–140 (15)	0.001^a^
	Obese	46	32.98 ± 27.04	11–117 (22)	
AST/ALT ratio	Non-obese	65	1.04 ± 0.48	0.24–2.48 (0.87)	0.001^a^
	Obese	46	1.36 ± 0.51	0.72–3.16 (1.21)	
MPV/PLT ratio	Non-obese	65	0.04 ± 0.01	0–0.08 (0.04)	0.726^a^
	Obese	46	0.04 ± 0.01	0.02–0.08 (0.04)	

**Table 4:** Comparison of age and laboratory parameters according to BMI. a: Mann-Whitney test (minimum-maximum/median); b: Student’s t-test (mean ± SD). P-values <0.05 were considered statistically significant. HOMA-IR = homeostatic model assessment for insulin resistance; QUICKI = quantitative insulin sensitivity check index; Total-C = total cholesterol; HDL-C = high-density lipoprotein cholesterol; LDL-C = low-density lipoprotein cholesterol; TG = triglyceride; TG/Glu = triglyceride/glucose index

	Obesity status	n	Mean ± SD	Minimum-Maximum (median)	P-value
Age (year)	Non-obese	65	28.29 ± 8.97	16–49 (27)	0.093^a^
	Obese	46	31.43 ± 9.99	16–57 (28.5)	
Glucose (mg/dL)	Non-obese	65	90.82 ± 8.37	75–119 (89)	0.019^a^
	Obese	46	94.24 ± 9.15	69–119 (94)	
Insulin (µIU/mL)	Non-obese	65	10.52 ± 4.62	2.43–28.03 (10.07)	0.001^a^
	Obese	46	16.47 ± 9.16	6.25–53.02 (14.22)	
HOMA-IR	Non-obese	65	2.33 ± 1.11	0–6.5 (2.25)	0.001^a^
	Obese	46	3.53 ± 2.38	0–12.9 (3.1)	
QUICKI	Non-obese	65	0.34 ± 0.03	0.29–0.43 (0.34)	0.001^a^
	Obese	46	0.32 ± 0.02	0.27–0.37 (0.32)	
Total-C (mg/dL)	Non-obese	65	182.65 ± 37.97	106–276 (182)	0.476^b^
	Obese	46	177.87 ± 29.26	123–269 (177.5)	
HDL-C (mg/dL)	Non-obese	65	55.14 ± 14.98	26–104 (53)	0.008^a^
	Obese	46	48.13 ± 14.61	28–82 (45.5)	
LDL-C (mg/dL)	Non-obese	65	103.81 ± 34.16	33–194 (103)	0.611^b^
	Obese	46	100.74 ± 26.06	45–172 (97)	
TG (mg/dL)	Non-obese	65	117.85 ± 96.91	38–665 (92)	0.012^a^
	Obese	46	137.52 ± 68.68	50–344 (126)	
TG/Glu	Non-obese	65	1.29 ± 1.03	0.47–7 (1.02)	0.023^a^
	Obese	46	1.47 ± 0.75	0.46–4.14 (1.29)	

We conducted a simple logistic regression analysis to find the independent predictors of obesity. We added the relevant parameters to the model. The BMP1, QUICKI values, and AST/ALT ratio were independent significant predictors (β = 1.066, p = 0.048; β = 0.0001, p = 0.001; and β = 3.707, p = 0.003; respectively) (Table 5). The multiple logistic regression analysis performed to determine the effect of independent variables on obesity was found to be statistically significant (chi-square = 29.961, p < 0.001). The independent variables in the model explained 23.7% of the total variance in obesity (p < 0.01). When the regression coefficients were examined, it was seen that the QUICKI (β = 0.001, p = 0.001) and the AST/ALT ratio (β = 2.803, p = 0.033) had a positive and significant effect on obesity (Table 5).

**Table 5:** Logistic regression analysis findings for the prediction of obesity with independent variables. P-values <0.05 were considered statistically significant. BMP1 = bone morphogenetic protein 1; AST/ALT = aspartate aminotransferase/alanine aminotransferase; QUICKI = quantitative insulin sensitivity check index; CI = confidence interval

Variables	Univariable					Multivariable				
	β	S. Fold	Exp (B) (95% CI)	Wald	P-value	β	S. Fold	Exp (B) (95% CI)	Wald	P-value
BMP1	0.064	0.032	1.066 (0.920–1.105)	3.897	0.048					
QUICKI	-43.444	10.95	0.0001 (0.0000–0.0002)	15.745	0.001	-40.293	11.07	0.001 (0.0000–0.0002)	13.229	0.001
AST/ALT ratio	1.310	0.438	3.707 (2.731–4.745)	8.958	0.003	1.031	0.483	2.803 (1.452–4.215)	-4.556	0.033

**Figure 1:**
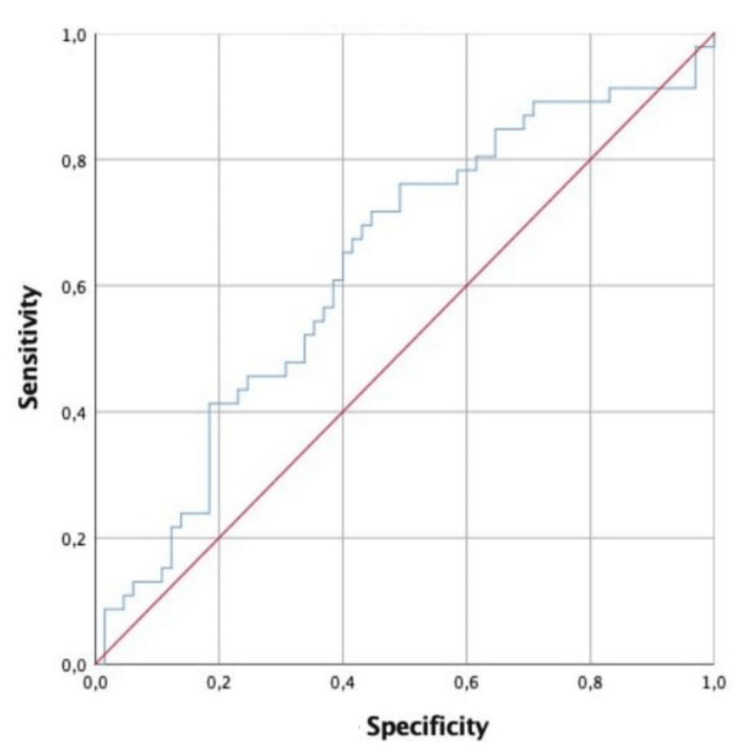
ROC analysis of BMP1. ROC = receiver operating characteristic; BMP1 = bone morphogenetic protein 1

The percentages of sensitivity and specificity at specific cut-off points of BMP1 derived from the coordinate of the ROC curve are shown in Figure 1. The cut-off value of BMP1 for predicting patients was 10.94 with 71.7% sensitivity and 55.4% specificity (area under the curve = 63.2, 95% confidence interval = 0.48-0.71; p < 0.001) (Figure 1).

## Discussion

In this study, we found that the independent variables, i.e., BMP1, QUICKI values, and AST/ALT ratio, were significantly related to obesity. Our study showed that the AST/ALT ratio had a significantly positive and QUICKI variables had a significantly negative effect on obesity. In other words, an increase in the AST/ALT ratio causes a predisposition to obesity, while a decrease in the QUICKI value causes a predisposition to obesity. In the obese participants, TG/Glu and AST/ALT ratios were significantly higher, while the QUICKI was lower than in the non-obese participants.

Obesity is correlated with type 2 DM, non-alcoholic fatty liver disease, hepatic steatosis, cardiovascular diseases, paralysis, dyslipidemia, hypertension, gallbladder diseases, osteoarthritis, sleep apnea, respiration problems, and some specific cancer types, and, together with such diseases, obesity might increase the death risk [23]. Mayoral et al. reported that there is a need to come up with better reagents to accurately distinguish the subtypes of obesity and reach an international consensus on terminology [24]. Multifactorial obesity involves several causes including environmental factors such as diet, lack of physical exercise, ultra-processed foods, fast food, microbiome, and chemical contaminants. Our study aimed to research the correlation between obesity and serum BMP1, NRG4, and ApoA5 levels.

ApoA1 is secreted as a proprotein. ApoA1 is cleaved by BMP1 and HDL-C is produced. Therefore, variations of the *BMP1* gene may affect serum ApoA1 and HDL-C levels. Teker et al. reported that BMP1 variation may affect the serum ApoA1 and HDL-C levels and thus contribute to the development of coronary heart disease (CHD) [25]. The study by Banerjee et al. identified that BMP1 regulates cellular LDL-C uptake [26]. These studies may explain the lower LDL-C concentrations. Unlike many studies in the literature about the correlation between BMP1, HDL-C, and LDL-C, our study proves to be significant, being the first to demonstrate the correlation between BMP1 and obesity. Our study showed that BMP1 levels in obese patients were higher than in non-obese patients, and BMP1 levels are an independent significant predictor of obesity on univariate analysis. On multivariate analysis, QUICKI and AST/ALT ratios were independent significant predictors of obesity. As our patient population was small, we think that the BMP1 level may not have predicted obesity.

The level of NRG4 in circulation was relatively high compared to prediabetic and diabetic patients and was an independent risk factor related to diabetes [27]. It led us to think that serum NRG4 concentrations might be a protective factor in the development of metabolic syndrome [27]. Wang et al. reported that adipose tissue NRG4 expression was decreased in obese animals and obese human adults, and low serum NRG4 concentrations were related to IR, obesity, and fatty liver disease in obese children [15,16]. In our study, even though it did not differ significantly, NRG4 levels were found to be lower in obese than the other groups, which supported the results of the above-mentioned study.

The ApoA5 level was low and inversely proportional to BMI in obese participants. ApoA5 decreased triglyceride levels by stimulating lipoprotein lipase activity in adipose tissue and modulated excessive triglyceride accumulation in the study by Su et al. [28]. Although statistical significance was not determined in this study, the ApoA5 levels were found to be lower in obese patients, which is consistent with the literature.

A study showed notable associations of ALT level and AST/ALT ratio with metabolic syndrome and its components, particularly fasting hyperglycemia and abdominal obesity among non-diabetics. As a significant result of the study, the ALT level and AST/ALT ratio were associated with the risk of prospective development of metabolic syndrome [29]. The TG/Glu ratio can be utilized as a surrogate indicator to define IR in healthy people. It can be a basic but effective surrogate biomarker to monitor the development of type 2 DM, metabolic syndrome, and carotid atherosclerosis. Furthermore, increased TG/Glu ratio was related to a high risk of prehypertension and hypertension [30]. QUICKI, as an index for IS acquired from a fasting blood sample, might prove useful for research and has been used extensively in studies. Thus, the remarkable results of our study have been that the AST/ALT and TG/Glu ratio of the obese were higher than those of the other group, while the QUICKI values were lower. Second, the AST/ALT ratio and the QUICKI values were independent significant predictors of obesity.

This study had several limitations. The cross-sectional design of this study is the most important limitation. The single-center trial and the limited number of patients are other limitations of the research. Finally, prospective molecular researches are required to determine the relationship between obesity and all the investigated markers.

## Conclusions

BMP1 levels, along with AST/ALT and TG/Glu ratios, were found to be significantly higher in obese patients and were an independent significant predictor of obesity together with AST/ALT ratio and QUICKI, suggesting that it may show a metabolic deterioration in obese individuals; however, it will be difficult to distinguish from these results whether it supported deterioration or was a component of the repair mechanism. Although BMP1 has been shown to play a role in osteogenesis in studies conducted to date, we obtained encouraging results suggesting that BMP1 might play a role in the pathogenesis of obesity. Thus, we expect that our results will promote the elucidation of the pathogenesis of obesity as well as come up with a therapeutic agent.
